# Sphingosine 1-phosphate axis: a new leader actor in skeletal muscle biology

**DOI:** 10.3389/fphys.2013.00338

**Published:** 2013-11-25

**Authors:** Chiara Donati, Francesca Cencetti, Paola Bruni

**Affiliations:** ^1^Dipartimento di Scienze Biomediche, Sperimentali e Cliniche, University of FlorenceFlorence, Italy; ^2^Istituto Interuniversitario di MiologiaItaly

**Keywords:** sphingosine 1-phosphate, skeletal muscle, skeletal muscle progenitors, satellite cells, muscle regeneration, insulin responsiveness, myoblasts, skeletal muscle metabolism

## Abstract

Sphingosine 1-phosphate (S1P) is a bioactive lipid involved in the regulation of biological processes such as proliferation, differentiation, motility, and survival. Here we review the role of S1P in the biology and homeostasis of skeletal muscle. S1P derives from the catabolism of sphingomyelin and is produced by sphingosine phosphorylation catalyzed by sphingosine kinase (SK). S1P can act either intracellularly or extracellularly through specific ligation to its five G protein-coupled receptors (GPCR) named S1P receptors (S1PR). Many experimental findings obtained in the last 20 years demonstrate that S1P and its metabolism play a multifaceted role in the regulation of skeletal muscle regeneration. Indeed, this lipid is known to activate muscle-resident satellite cells, regulating their proliferation and differentiation, as well as mesenchymal progenitors such as mesoangioblasts that originate outside skeletal muscle, both involved in tissue repair following an injury or disease. The molecular mechanism of action of S1P in skeletal muscle cell precursors is highly complex, especially because S1P axis is under the control of a number of growth factors and cytokines, canonical regulators of skeletal muscle biology. Moreover, this lipid is crucially involved in the regulation of skeletal muscle contractile properties, responsiveness to insulin, fatigue resistance and tropism. Overall, on the basis of these findings S1P signaling appears to be an appealing pharmacological target for improving skeletal muscle repair. Nevertheless, further understanding is required on the regulation of S1P downstream signaling pathways and the expression of S1PR. This article will resume our current knowledge on S1P signaling in skeletal muscle, hopefully stimulating further investigation in the field, aimed at individuating novel molecular targets for ameliorating skeletal muscle regeneration and reducing fibrosis of the tissue after a trauma or due to skeletal muscle diseases.

## Introduction on skeletal muscle

Skeletal muscle represents about 40% of the entire body weight. The functional and structural constituent of skeletal muscle is the myofiber, multinucleated cell that derives from the fusion of mesodermal precursors named myoblasts. Each myofiber is delimited by the basal lamina and is composed of myofibrils of actin and myosin, organized as a sarcomere, the functional unit of skeletal muscle. On the basis of their physiological features, muscle fibers can be distinguished into slow contracting, fatigue resistant fibers and fast contracting, fatigue sensitive fibers.

Skeletal muscle exerts physiological roles relevant for the control of body metabolism since it accounts for bloodstream aminoacid disposal in fasting condition and for removing excess glucose by favoring glycogen accumulation in response to insulin after a meal. Moreover, by releasing heat during force generation it contributes to body temperature maintenance.

During development, the number of myofibers following myoblast fusion increase, while in adulthood skeletal muscle is stable beyond infrequent fusion of satellite cells to compensate physiologic turnover. Following a trauma, injury, or disease skeletal muscle has indeed the potential to regenerate thanks to an highly orchestrated process driven by satellite cells and their niche (Yin et al., 2013).

Satellite cells are the resident stem cells of the skeletal muscle, located between the basal lamina of muscle fibers and the plasma membrane. Even if it is known that satellite cells derive from somites, their exact progenitor cell is still elusive. Satellite cells are quiescent in adult skeletal muscle and represent 3–6% of the total nuclei in the fiber (Tedesco et al., 2010). When activated, they start proliferating and can generate in few days a large number of myofibers: it has been demonstrated that few as seven satellite cells transplanted into irradiated muscle of dystrophic immune-deficient mice led to an increase of 100 new muscle fibers with thousands of myonuclei (Collins et al., 2005). Activated satellite cells cultured on plastic collagen-coated dishes are named “satellite cell-derived myoblasts” or myogenic precursor cells distinct from a functional and molecular point of view from freshly isolated satellite cells, possibly due to the absence of their niche (Dhawan and Rando, 2005; Pallafacchina et al., 2010). Stem cells can self-renew in asymmetric and symmetric division. In asymmetric division the parental stem cell divides into two different daughter cells, one remains stem cell, the other fated to differentiate. In symmetric division the parental stem cell gives rise to two daughter stem cells of equal stemness. Satellite cells can undergo asymmetric and symmetric division and non-canonical Wnt signaling plays a role in the regulation of self-renewal (Chargé and Rudnicki, 2004; Troy et al., 2012). A number of satellite cell markers have been identified such as Pax3, Pax7, Myf5, M-cadherin, cMet, CD34, CXCR4, although they are not unique and there is not complete correspondence between mouse and human (Yin et al., 2013).

In addition to satellite cells, other skeletal muscle progenitors including pericytes (Dellavalle et al., 2011), mesoangioblasts (Minasi et al., 2002), bone marrow stem cells (LaBarge and Blau, 2002), and interstitial cells (Mitchell et al., 2010) located outside the myofiber, have been demonstrated to have the ability to regenerate skeletal muscle, although to a negligible extent compared with satellite cells (Judson et al., 2013). Nowadays there is a great interest in understanding the molecular mechanisms of skeletal muscle regeneration since it will permit the development of cell therapies for skeletal muscle diseases such as muscular dystrophies (Tedesco et al., 2010), a group of inherited disorders characterized by progressive muscle wasting and depletion of the satellite cell pool after extensive cycle of regeneration (Carlson and Conboy, 2007; Chakkalakal et al., 2012; Pannérec et al., 2012; García-Prat et al., 2013). Muscular dystrophies are indeed very difficult to treat because skeletal muscle is composed of hundreds of millions of post-mitotic nuclei.

## Sphingolipid metabolism and sphingosine 1-phosphate formation

During the last 20 years, sphingolipids, initially considered structural component of eukaryotic membranes, have been highlighted as a crucial molecules involved in the regulation of fundamental biological processes such as cell migration, survival, proliferation, differentiation, adhesion, and implicated in inflammation, tumorigenesis, immunity and vascular development (Hannun and Obeid, 2008; Maceyka et al., 2012). In this review we will focus on the role of the bioactive sphingolipid sphingosine 1-phosphate (S1P), its metabolism, its receptors and its cross-talk with growth factors and cytokines in skeletal muscle biology and physiology.

Ceramide, a largely known effector of stress responses (Zeidan and Hannun, 2010) plays a central role in sphingolipid metabolism. The first step in the *de novo* pathway of ceramide production is the condensation reaction catalyzed by the enzyme serine palmitoyl transferase to form dihydrosphingosine from palmitoyl-CoA and the aminoacid serine. Dihydrosphingosine is then acylated by the action of ceramide synthase to dihydroceramide which is then desaturated to ceramide by the enzyme ceramide desaturase. Ceramide can be also generated by the breakdown of membrane sphingomyelins, catalyzed by the action of various sphingomyelinases or by the degradation of complex glycosphingolipids by the action of glucosylceramidases. The acyl chain of ceramide is then removed by the action of ceramidases and the amino alcohol sphingosine is produced. Sphingosine can be reconverted to ceramide by the action of ceramide synthase via the so-called salvage pathway mechanism. Sphingosine can be phosphorylated in an ATP-dependent manner to S1P by the enzymes sphingosine kinase (SK) 1 and SK2. S1P can be dephosphorylated back to sphingosine by the action of two specific S1P phosphatases and by three lipid phosphate phosphatases. Alternatively, S1P is irreversibly degraded by the action of S1P lyase (SPL) into hexadecenal and phosphoethanolamine.

SK1 is a cytosolic enzyme which contains residues that bind acidic phospholipids that contribute to its intracellular localization (Stahelin et al., 2005). Numerous agonists have been reported to be able to activate SK1, including growth factors, hormones, cytokines, and G protein-coupled receptors (GPCR) ligands. Following ERK phosphorylation at serine 225, SK1 translocates to plasma membrane where its substrate sphingosine is localized (Pitson et al., 2003). The regulation of SK localization within the cell is the major mechanism by which the enzyme affects sphingolipid metabolism since only minute increases in the activity are reported following stimulation with different agonists. Once produced, S1P, precisely partitioned into plasma membrane microdomains, is then locally released to activate S1P receptors (S1PR) acting in autocrine and/or paracrine manner. This process is called “inside-out” signaling. Different transporters have been implicated in S1P export such as the ATP binding cassette transporters, ABCC1 (Mitra et al., 2006), ABCA1 (Sato et al., 2007), ABCG2 (Takabe et al., 2010), and more recently the specific spinster 2 (Spns2) (Kawahara et al., 2009); however, no information on their role in skeletal muscle is presently available. Additionally, since the chloride ion channel CFTR has a role in mediating S1P transport and signaling in heart (Meissner et al., 2012) and lack of CFTR causes functional alteration in skeletal muscle (Divangahi et al., 2009), it will be of interest to further investigate this issue.

In contrast to SK1, SK2 is localized in several intracellular compartments such as endoplasmic reticulum, nucleus, and mitochondria. Even if it is known that ERK-mediated phosphorylation is required for SK2 activation, the exact mechanism of SK2 regulation is still elusive. While SK1-formed S1P is rapidly exported outside the cell through the transporters localized at the plasma membrane, SK2-produced S1P at the level of mitochondria and endoplasmic reticulum is likely rapidly degraded or dephosphorylated by SPL and phosphatases present in close proximity. Therefore, SK2 has, in respect to SK1, an enhanced ability to recycle sphingoid bases for ceramide synthesis (Le Stunff et al., 2007). Compared to SK1, less is known about the mechanism of SK2 regulation. It has been reported that EGF and phorbol ester (Hait et al., 2007) activates the enzyme. Moreover, under hypoxia an increase in SK2 protein level and enzymatic activity has been demonstrated (Schnitzer et al., 2009).

Bioactive S1P can either function as ligand of a family of GPCR named S1PR and through intracellular targets, some of them recently identified. There are five specific S1PR, S1PR_1−5_, all with low nM Kd values. Since these receptors couple to multiple α subunits of heterotrimeric G proteins and are differentially expressed in diverse cell types and tissues, they induce the activation of different downstream targets such as ERK, Rac, Rho, JNK, adenylyl cyclase, phospholipase C, thus evoking distinct, sometimes overlapping or opposite, biological responses. While S1P_1−3_ are ubiquitously expressed, S1P_4_ and S1P_5_ are tissue specific, being mostly expressed in the lymphoid and central nervous system, respectively (Spiegel and Milstien, 2003). S1P_1_ plays a crucial role in angiogenesis, indeed its deletion in mice is embryonic lethal due to hemorrhage for incomplete vascular maturation since pericytes do not migrate to the nascent endothelial tubes (Liu et al., 2000). S1P_2_ null mice are deaf indicating that S1P_2_ is required for proper development of the auditory and vestibular systems (Kono et al., 2007). Unlike S1P_1_ null mice, S1P_3_ deletion in mice do not generate any obvious phenotype.

A number of cytokines and growth factors have been reported to regulate the expression of the enzymes of S1P metabolism (Lebman and Spiegel, 2008). In addition to S1PR transactivation following stimulation with growth factors and cytokines, S1P ligation to its receptors also transactivates growth factor tyrosine kinase receptors. This mutual functional cross-talk regulates fundamental biological processes such as growth, differentiation, and motility in various cell types.

Many data reported in literature support that the intracellular role of S1P is that of counteracting the biological actions of ceramide in the so-called “rheostat model” where the S1P-forming enzyme SK plays a crucial role. Although S1P was discovered as intracellular messenger (Olivera and Spiegel, 1993), only recently, some of the intracellular targets of S1P have begun to be identified. SK2 present in the nucleus has been demonstrated to form a complex with histone H3 and histone deacetylases (HDACs). SK2-produced S1P binds to and inhibits HDAC1/2, thus contributing to the regulation of the epigenetic control of specific genes (Hait et al., 2009). Instead, tumor necrosis factor receptor associated factor 2 (TRAF2), a crucial component in NFkB signaling triggered by TNFα, has been reported as intracellular target for S1P produced by SK1. Indeed, S1P formed by SK1, which is known to co-localize with TRAF2 (Xia et al., 2002) has been found to bind to TRAF2 and stimulate its dormant ubiquitin ligase activity upstream of NFkB activation (Alvarez et al., 2010). Moreover, SK2-produced S1P in the mitochondrion binds *in vitro* and *in vivo* to prohibitin 2, a conserved protein that regulates mitochondrial assembly and function. SK2 null mice display reduced mitochondria respiration, suggesting that S1P/prohibitin 2 interaction is physiologically relevant for mitochondrial function (Strub et al., 2011).

## Biological role of S1P in satellite cells

A number of recently published papers support the role of S1P axis in the regulation of muscle resident satellite cell biology. Indeed, S1P was identified as one of the few extracellular cues capable of stimulating quiescent satellite cells to enter the cell cycle (Nagata et al., 2006). Inhibition of S1P formation by incubation with the SK inhibitor dimethylsphingosine (DMS) significantly reduced satellite cell activation in response to mitogen and impaired satellite cell-driven muscle regeneration in response to *in vivo* damage induced by cardiotoxin. Moreover, the authors showed that the degradation of the sphingomyelin inner leaflet pool accounts for the generation of S1P, then regulating satellite cell activation. In line with these findings, Calise et al. have characterized the S1PR involved in the mitogenic effect of S1P and the underlying mechanism of action (Calise et al., 2012). Satellite cells express 4 out of 5 S1PR, S1PR_1−4_, being S1P_3_ the most expressed one in growing conditions. Indeed, by utilizing specific receptor antagonist and siRNA silencing, S1P was demonstrated to stimulate labeled thymidine incorporation by engagement of S1P_2_ and S1P_3_ in a PI3K-dependent manner. Moreover, the authors demonstrated also that S1P positively stimulates satellite cell migration via specific ligation to S1P_1_ and S1P_4_. This latter finding, for the first time, highlights the role of S1P_4_ in skeletal muscle beyond its already established relevance in lymphoid tissue. Very recently it has been shown that S1P_3_ suppresses cell cycle progression in murine satellite cells (Fortier et al., 2013). Indeed, satellite cells isolated from S1P_3_ null mice showed enhanced *ex-vivo* proliferation, while retrovirally-mediated constitutive expression of S1P_3_ inhibited cell proliferation of satellite cells.

## Biological role of S1P in other skeletal muscle progenitors

Skeletal muscle regeneration, besides being due to the presence of resident satellite cells in skeletal muscle, is carried on, *in vivo* or after transplantation, by progenitors that originates outside the basal lamina such as pericytes, interstitial cells, mesoangioblasts, and adipose tissue-derived mesenchymal stem cells (ASC). The characterization of the molecular mechanism of the myogenic potential of these progenitors is nowadays of great interest since such kind of knowledge could be applied in the development of cell therapies for diseases characterized by skeletal muscle degeneration. S1P axis has been shown to play a role in the regulation of fundamental biological parameters such as proliferation, migration and differentiation of multipotent adult stem cells such as mesoangioblasts (Donati et al., 2007a, 2009, 2011), and ASC (Nincheri et al., 2009).

Mesoangioblasts (Minasi et al., 2002) are vascular progenitors associated with dorsal aorta in avian and mammalian species, that when transplanted *in vivo* give rise to multiple mesodermal cell types such as osteoblasts, chondrocytes, and muscle cells. They show an extensive ability of *in vitro* self-renewing and upon injection, are able to cross the endothelial barrier and can fuse with muscle fibers, contributing to their regeneration. Indeed, mesoangioblasts have been demonstrated to contribute to muscle regeneration in animal models of muscular dystrophy such as α-sarcoglycan null mice (Sampaolesi et al., 2003) and golden retriever dystrophic dogs (Sampaolesi et al., 2006). S1P_1−3_ were detected at mRNA level in mesoangioblasts with S1P_3_ the predominantly expressed receptor. S1P has been demonstrated to potently stimulate mesoangioblast proliferation in S1P_2_-dependent manner (Donati et al., 2007a). Moreover, the bioactive sphingolipid successfully contrasted the apoptosis induced by different apoptogenic stimuli, without engagement of S1PR. It is important to note that pre-treatment of mesoangioblasts with S1P enhanced their survival when injected in the tibialis anterior muscle of α-sarcoglycan null dystrophic mice, further supporting a role for the sphingolipid in preventing programmed cell death. Moreover, the SK/S1P axis was found to be involved in the anti-apoptotic action exerted by TGFβ in these cells (Donati et al., 2009). RNA silencing or overexpression of dominant negative mutant form of SK1 highlighted a key role of SK1 but not SK2 in mediating TGFβ pro-survival action. The cytokine increased SK activity and up-regulated SK1 expression which was down-regulated by the apoptotic challenge. A successive study demonstrated the crucial role of the cross-talk between S1P and TGFβ in the biology of mesoangioblasts. Data obtained by a cDNA microarray study, validated by real time PCR and immunofluorescence microscopy approaches, highlighted that S1P exerts a strong differentiating action of mesoangioblasts toward smooth muscle cells. Interestingly it was shown that S1P axis was required by TGFβ to exert its differentiating activity toward smooth muscle on mesoangioblasts. The study individuated also the transcription factor GATA6 as a novel player in the complex transcriptional regulation of mesoangioblast differentiation to smooth muscle. S1P up-regulated the expression of GATA6 which was responsible for the enhanced expression of smooth muscle contractile proteins. By specific silencing of GATA6 it was demonstrated that the transcription factor was critical also in the pro-differentiating activity of TGFβ (Donati et al., 2011).

ASC are mesenchymal stem cells able to differentiate into different mesodermal lineages (Chamberlain et al., 2007). Being relatively easy to isolate and able to rapidly proliferate *in vitro*, these cells are promising in the development of cell therapy tools. S1P administration to ASC up-regulated the expression of smooth muscle protein markers, induced the appearance of calcium currents and of actin cytoskeleton reorganization typical of smooth muscle cells. S1P differentiating action was found to rely on S1P_2_ engagement while S1P_3_ role was less critical (Nincheri et al., 2009).

## Biological role of S1P signaling axis in myoblasts

C2C12 cell line is a subclone of the parental C2 cells which differentiate into myotubes in appropriate culture condition, after serum deprivation (Yaffe and Saxel, 1977; Blau et al., 1985). This model is therefore widely used as a tool to study the myogenic differentiation process and the involved molecular mechanisms.

C2C12 cells express 4 out of 5 S1PR, S1P_1−4_, being S1P_1_ the most expressed receptor (Meacci et al., 2003; Bruni and Donati, 2013; Donati et al., 2013). Earlier reports showed that in C2C12 cells exogenous S1P activates a number of signaling pathways: indeed it induced a rapid stimulation of phospholipase D activity (Meacci et al., 1999) and a transient and rapid membrane association of RhoA (Meacci et al., 2000) in a PKC-dependent manner. Moreover, the bioactive lipid was demonstrated to induce Ca^2+^ mobilization in a S1P_2/3_-dependent manner (Meacci et al., 2002). Interestingly, differentiation of C2C12 myoblasts into myotubes was accompanied by deep changes in expression of S1PR. Indeed, S1P_2_ was down-regulated while S1P_3_ was up-regulated in differentiated cells (Meacci et al., 2003), suggesting that S1P_2_ plays a role in myoblasts but is dispensable in myotubes. The crucial role of S1P_2_ in myogenic differentiation of C2C12 myoblasts was demonstrated in a later study, where S1P was shown to exert via this receptor subtype a negative action on serum-induced proliferation and to act as a potent inducer of differentiation (Donati et al., 2005). The role of S1P_2_ was demonstrated by pharmacological and genetic approaches. The involvement of S1P_2_ in myogenic differentiation was further confirmed by a subsequent report where the compound K6PC5, a synthetic derivative of ceramide, previously shown to induce SK1 activation in keratinocytes (Kwon et al., 2007), stimulated myoblast differentiation in a S1P_2_-dependent manner (Bernacchioni et al., 2011).

S1P_2_ was also identified as the responsible for the inhibitory effect on myoblast directional motility and insulin like growth factor-1 (IGF-1)-induced chemotaxis exerted by S1P in C2C12 myoblasts (Becciolini et al., 2006), in line with the anti-migratory action of this receptor demonstrated in other cell systems (Arikawa et al., 2003; Lepley et al., 2005). In this study was also established the involvement of RhoA activation in the negative regulation of cell motility.

During muscle regeneration, following satellite cell activation and migration to the site of lesion, inhibition of myoblast motility and differentiation into myotubes takes place in a highly orchestrated manner. In view of its role as muscle pleiotropic regulatory molecule, S1P exerts a dual role on cell migration, stimulating at first migration of activated satellite cells (Calise et al., 2012) and then inhibiting that of C2C12 myoblasts (Becciolini et al., 2006), subsequently to a timely remodeling of S1PR expression pattern, thus favoring skeletal muscle repair (Table 1).

**Table 1:**
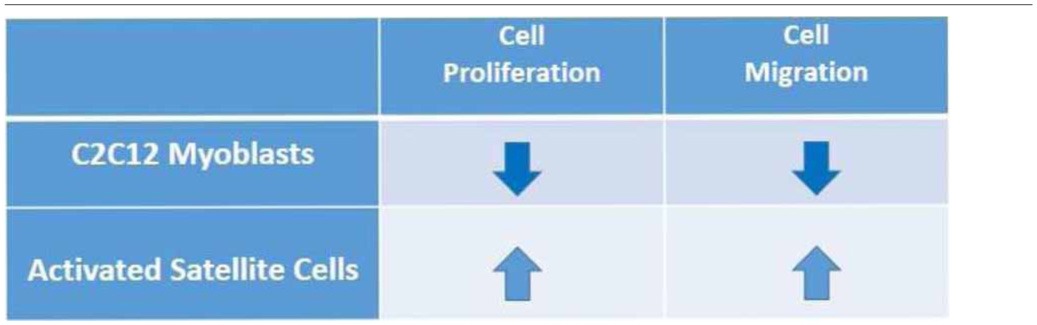
**Role of sphingosine 1-phosphate on cell proliferation and migration in myoblasts and activated satellite cells**.

During *in vitro* differentiation of C2C12 myoblasts, a small population of cells, named reserve cells remains undifferentiated, thus resembling quiescent satellite cells (Yoshida et al., 1998). The analysis of the role of S1PR in regulating cell proliferation highlighted that in reserve cells, S1P, via specific coupling to S1P_1_, stimulates cell proliferation similarly to what demonstrated in satellite cells but differently from what shown in myoblasts (Rapizzi et al., 2008). In view of the finely regulated expression of S1PR during commitment and differentiation of myogenic precursors which drive different biological outcomes of the bioactive lipid, future studies are required to characterize the specific molecular mechanisms regulating the differential expression of S1PR and their coupling.

Successive studies have elucidated some downstream events implicated in the promyogenic effect of the sphingolipid. Indeed, exogenous addition of S1P to C2C12 myoblasts up-regulated the expression of the gap junctional protein connexin-43 (Squecco et al., 2006), and the transient receptor potential cation channel, a component of the stretch activated channel (Formigli et al., 2007; Meacci et al., 2010).

The role of S1P metabolism, with special attention to SK, the enzyme responsible for S1P production, in the regulation of C2C12 cell growth and differentiation has been highlighted. Indeed, the expression of SK1 was found to be enhanced in differentiating myoblasts (Meacci et al., 2008). Additionally, when overexpressed, SK1 was responsible for a significative reduction of myoblast proliferation rate, while it enhanced the appearance of a differentiated phenotype and the expression of myogenic marker proteins. On the contrary, when SK1 was silenced or a dominant negative mutant form of the enzyme was overexpressed, myoblast proliferation was increased and myogenic differentiation rate was reduced. The role of S1P_2_ in myogenic differentiation was also confirmed in this study, since when the receptor was silenced, the pro-myogenic action of SK1 overexpression was abolished. Therefore, although in the literature SK1 has been reported to have a mitogenic role (Maceyka et al., 2012), in myoblasts this enzyme displays an opposite biological effect.

**Figure 1:**
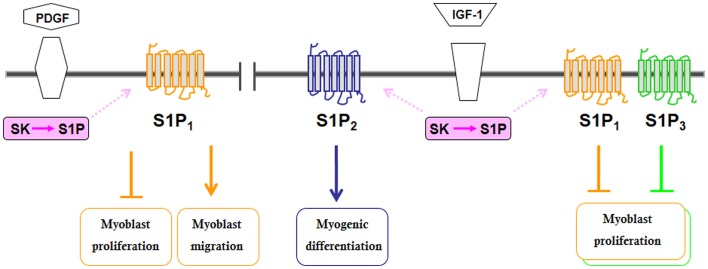
**Schematic diagram of the biological actions evoked by S1PR transactivation by some growth factors in C2C12 myoblasts**.

**Figure 2:**
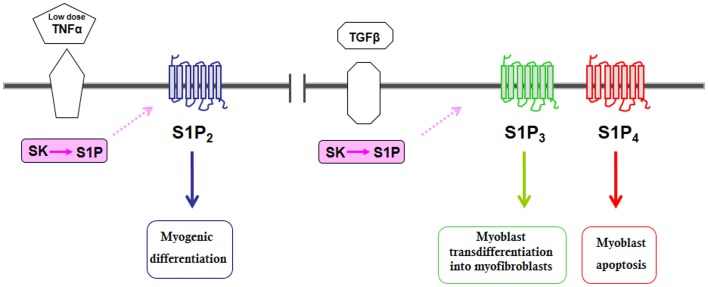
**Schematic diagram of the biological actions evoked by S1PR transactivation by some cytokines in C2C12 myoblasts**.

As outlined above, SK activity is under the control of a variety of growth factors, cytokines, neurotransmitters, and hormones (Maceyka et al., 2012), which leads to the existence of functional cross-talks further complicating S1P signaling. Following the demonstration of the role of SK1 in myogenesis, several reports have shown that a number of growth factors and cytokine, known as crucial regulator of skeletal muscle biology, co-opt S1P signaling (Figures 1, 2). The pro-inflammatory cytokine TNFα, which is critically implicated in the remodeling of skeletal muscle (Li and Schwartz, 2001), at low doses has been shown to translocate SK1 to membrane and, via S1P_2_ engagement, to stimulate C2C12 myoblast differentiation (Donati et al., 2007b). Notably, IGF-1, one of the most important physiological regulator of skeletal muscle regeneration (Pedersen and Febbraio, 2012), activates both SK isoforms, SK1 and SK2, and via specific transactivation of S1P_2_, exerts its myogenic action (Bernacchioni et al., 2012). Moreover, IGF-1 is also responsible for SK-dependent transactivation of S1P_1_ and S1P_3_ that in turn reduce the mitogenic effect of the growth factor.

Interestingly, also platelet derived growth factor (PDGF), another growth factor relevant for the biology and the repair of skeletal muscle (Husmann et al., 1996), exerts a negative role on myoblast proliferation via selective SK1 activation and S1P_1_ transactivation. Moreover, S1P inside-out signaling and S1P_1_ engagement appear to be necessary for conveying of PDGF-induced chemotactic action. It is important to note that despite the importance of S1P_1_ engagement in the biological action of IGF-1 and PDGF in myoblasts, S1P_1_ down-regulation or pharmacological inhibition did not affect the biological responses evoked by exogenously added S1P (Donati et al., 2005; Becciolini et al., 2006). This observation suggests that spatial regulation of S1P generation inside the cell is critical for determining the subset of engaged receptors, its final biological outcomes and that S1P_1_ is not freely accessible to its ligand from outside the cell. Indeed, structural evidence indicated that ligand binding to S1P_1_ occurs by initial delivery of S1P to the exterior portion of membrane followed by a lateral diffusion into the receptor binding pocket (O'Sullivan and Dev, 2013).

SK/S1P axis is also exploited by TGFβ to transmit its pro-fibrotic, anti-differentiating action in C2C12 myoblasts (Cencetti et al., 2010). Unfortunately, during muscle regeneration, the transdifferentiation of myoblasts and muscle resident fibroblasts into myofibroblasts give rise to muscle fibrosis that reduces muscle contractile properties and impairs full muscular recovery. The cytokine TGFβ plays a crucial role in the promotion of the fibrosis onset and in the inhibition of myogenesis (McLennan and Koishi, 2002); importantly, the inhibition of TGFβ signaling pathway has been shown to ameliorate the chronic degenerative fibrosis of dystrophic muscles (Cohn et al., 2007). Indeed, TGFβ induced a Smad-dependent up-regulation of SK1 expression and activation, which, in contrast to what expected on the basis of the previously demonstrated SK1 role in myogenesis (Donati et al., 2005; Meacci et al., 2008), redirected the final S1P biological outcome from being promyogenic to profibrotic since the cytokine induced also a profound remodeling of S1PR expression pattern, at least at mRNA levels. This was responsible for readdressing the pro-myogenic role mediated through S1P_2_ to a pro-fibrotic role mediated by S1P_3_, which following TGFβ administration, became the most expressed receptor. These data support the concept that interfering with S1P_3_ signaling might favor therapeutic intervention to reduce skeletal muscle fibrosis.

Very recently a new signaling pathway related to TGFβ-induced apoptosis has been identified in C2C12 myoblasts which relies on Rho kinase-2 stimulation, subsequent to SMAD-dependent TGFβ-induced S1P_4_ up-regulation and transactivation via SK2 (Cencetti et al., 2013). This finding supports the notion that S1P_4_ is up-regulated by TGFβ and is relevant for the apoptotic action of the cytokine in skeletal muscle.

## Biological role of S1P in skeletal muscle

Skeletal muscle has the ability to adapt to different physiological and pathological conditions since it displays a high degree of plasticity. The size of skeletal muscle fibers results from a balance between protein synthesis and degradation, and varies as a consequence of physiological and pathological circumstances. The increase of individual muscle fibers, i.e., hypertrophy, occurs when protein synthesis exceeds protein degradation in response to hormonal stimulation or mechanical overload. A reduction in skeletal muscle mass and fiber size, i.e., atrophy, occurs in response to catabolic hormonal stimulation, denervation, aging, cancer, bed rest, and starvation (Schiaffino et al., 2013). S1P has been reported to be involved in the regulation of some physiological properties of skeletal muscle such as muscle contractility, fatigue, and adaptation.

Indeed, S1P has been shown to affect the excitation-contraction coupling in fibers isolated from murine extensor digitorum longus (EDL). The bioactive lipid modulated the voltage threshold and the inward calcium current through the dihydropyridine receptor in a S1P_3_-dependent manner although the experiments were carried out in the presence of the unspecific receptor antagonist suramin (Bencini et al., 2003).

Zanin et al. (2008) has demonstrated a trophic role for S1P in a rat model of denervation atrophy. S1P, delivered to the muscle through a mini osmotic pump, significantly reduced the negative effects of denervation on muscle mass and cross sectional area (CSA) after 7 and 14 days of regeneration. Moreover, despite its established anti-proliferative and pro-apoptotic role (Bruni and Donati, 2008), also sphingosine exerted a trophic effect, likely due to its conversion to S1P catalyzed by SK, as suggested by the abolishment of the positive action of the sphingolipid in the presence of DMS. Moreover, by real time PCR, S1P_1_, and S1P_3_ were found to be selectively expressed, and subjected to down-regulation upon denervation.

The decline in the ability of skeletal muscle to generate force after a strenuous exercise is named muscle fatigue. The process is reversible and depends on multiple factors such as the intensity and the duration of the contraction and the type of fibers of the muscles. Exogenous S1P has been found able of reducing muscle fatigue of EDL muscles (Danieli-Betto et al., 2005). Interestingly, administration of sphingosine significantly diminished the tension decline. Since sphingosine effect was reduced in the presence of the unselective SK inhibitor DMS, it was deduced that the mechanism of action implicates S1P production through SK activation. Indeed the effect of sphingosine, but not that of S1P, was abolished in the absence of Ca^2+^, suggesting a Ca^2+^-dependent mechanism of activation of SK.

The role of S1P signaling in skeletal muscle regeneration has been firstly demonstrated in mouse and rat models after myotoxic injury induced by bupivacaine (Danieli-Betto et al., 2010). The administration of S1P after muscle damage induced a significant increase of CSA while the neutralization of circulating S1P by administration of an anti-S1P antibody abolished fiber growth. Western blotting analysis of S1PR expression in muscle lysates during rat soleus regeneration showed dynamic changes of S1PR at protein level suggesting their putative role during regeneration. The S1P-induced increase of regenerating fiber growth was inhibited by a selective S1P_1_ agonist and augmented by a selective S1P_1/3_ antagonist, supporting a negative role of S1P_1_ and a positive role of S1P_3_ in the early phases of regeneration. In contrast, a very recent study demonstrated that in S1P_3_ null mice acute muscle regeneration was enhanced and that genetic ablation of S1P_3_ in mdx mice produced a less severe dystrophic phenotype (Fortier et al., 2013). Further studies are therefore required to clarify the exact role of S1P_3_ in skeletal muscle regeneration.

Herr et al. (2003) have demonstrated that mutation in the gene that encodes for the homolog of SPL generates Sply mutants in fruit fly that do not catalyze S1P degradation and accumulate S1P. In these mutants the thoracic flight muscles degenerate due to the absence of fibers in the dorsal longitudinal muscles. These findings highlight the importance of sphingolipid metabolism in muscle homeostasis, crucial for adult muscle development and integrity.

Presently, beside mammalian models to study muscular dystrophy including mouse, dog and cat, genetically tractable models have been established in *Danio rerio* and *Drosophila melanogaster* (Shcherbata et al., 2007). Very recently, a paper published by Pantoja et al. (2013), demonstrated that suppression of *wunen*, an homolog of lipid phosphate phosphatase 3, suppresses dystrophic muscle phenotype measured by evaluation of correct localization of the titin-homolog, projectin, in sarcomeres and functional movements. The authors demonstrated that wunen suppression acts through the elevation of S1P levels, since analogous findings were obtained altering S1P levels genetically via down-regulation of SPL or up-regulation of serine palmitoyl-CoA transferase, or pharmacologically by oral administration of SPL inhibitors. The same authors have very recently demonstrated that an increase of S1P levels via administration of an SPL inhibitor positively affected acute muscle regeneration of dystrophic mdx mice. The beneficial effects of S1P on increased muscle fiber size, force, diminished fibrosis, and fat accumulation were linked to the ability of S1P of increasing the number of myogenic cells (Ieronimakis et al., 2013).

The role of S1P_2_ *in vivo* in the early phases of regeneration has been demonstrated recently by Germinario et al. (2012) after injection of notexin in two mouse model of S1P_2_ deficiency: the S1P_2_ null mice and wild-type mice systemically administered with the S1P_2_ antagonist JTE-013. Indeed, the absence of S1P_2_ or its blockade delayed the regeneration of skeletal muscle measured as soleus CSA. Interestingly, the systemic administration of an anti-S1P antibody, which induced a reduction of soleus fiber growth in wild-type mice was ineffective in the absence of S1P_2_.

Microarray study revealed that during mouse skeletal muscle regeneration following notexin injury, while *sphk1* gene is upregulated at early time points, *sgpl1* gene is induced several days post injury (Loh et al., 2012). SPL is expressed at very low level in skeletal muscle at rest, while is up-regulated in response to ischemia (Kumar and Saba, 2009) and radiation (Bandhuvula et al., 2011). The specific requirement for SK1 to support muscle regeneration and satellite cell recruitment has been demonstrated in SK1 null mice. Interestingly the plasma of dystrophic mdx mice display reduced S1P levels, comparable to that of SK1 null mice, probably due to a significant up-regulation of *sgpl1* rather than down-regulation of *sphK1* or *sphK2* genes. Tetrahydroxybutylimidazole, unspecific inhibitor of SPL, improved the number of regenerating fibers in dystrophic muscles and satellite cell activation. The combination of pharmacological inhibitors, S1PR antagonists and plasmid constructs *in vitro* showed that S1P recruits satellite cells through activation on S1PR_2_/Rho GTPase/STAT3 signaling axis (Loh et al., 2012).

## Biological role of S1P in insulin responsiveness of skeletal muscle

Skeletal muscle plays a crucial role in the regulation of whole body metabolism. In response to insulin, in fed condition, skeletal muscle is responsible for glucose removal from the bloodstream thus contributing to the majority of glucose disposal for the building-up of glycogen. Since skeletal muscle utilizes the majority of body glucose, reduced insulin responsiveness in skeletal muscle leads to the development of metabolic syndrome (McGarry, 2002). SK1/S1P axis has recently been implicated in the regulation of glucose metabolism. In C2C12 myoblasts pharmacological inhibition of SK1 reduced insulin-stimulated glucose uptake while its overexpression mimicked *in vivo* insulin action. Moreover, overexpression of SK1 gene reduced blood glucose level in diabetic mice (Ma et al., 2007). In keeping with the role of S1P as insulin-mimetic cue, a study has reported that in C2C12 myoblasts S1P, through engagement of S1P_2_, produces a transient burst of reactive oxygen species which is sensed by protein tyrosine phosphatase-1B, the main negative regulator of insulin receptor phosphorylation, which undergoes inhibition. This blockade provokes a ligand-independent trans-phosphorylation of insulin receptor and a strong increase in glucose uptake (Rapizzi et al., 2009). This study appears to provide the possible mechanistic explanation of the insulin-mimetic action of SK1 overexpression, highlighting a feedback responsible for the sustained activation of insulin receptor. Since in this study possible implications of S1P-directed insulin signaling in diabetes were not established, future studies are required aimed at clarifying whether impaired S1P signaling contributes to the development of insulin resistance and whether improvement of S1P/S1P_2_ signaling would be beneficial for glucose disposal by skeletal muscle.

In addition to the role of elevated triacylglycerol levels in skeletal muscle for the development of insulin sensitivity (McGarry, 2002), a number of reports support the role of ceramide for diminished insulin responsiveness. Taking into account that SK plays a crucial role in the regulation of the relative levels of ceramide/sphingosine and S1P, it will be of crucial interest in the future to understand whether the biological effects observed as consequence of SK overexpression are due to specific S1P-induced signaling or rather to diminution of ceramide cellular levels.

In this line, transgenic mice overexpressing SK1 display improved insulin sensitivity compared to wild-type animal after a 6 week high fat diet, due to increased SK activity in skeletal muscle and decreased intracellular ceramide content (Bruce et al., 2012). The same authors have demonstrated that daily treatment with FTY720, that acts as functional antagonist of S1PR and has been reported to inhibit the activity of ceramide synthase *in vitro* (Berdyshev et al., 2009; Lahiri et al., 2009), for 6 weeks after an high fat diet improved mice glucose tolerance, insulin uptake and Akt phosphorylation through ceramide, diacylglycerol, and triacylglycerol reduction in muscle (Bruce et al., 2013). Interestingly, the expression levels of the enzymes involved in sphingolipid metabolism were not altered.

It has been demonstrated that treatment with palmitate in C2C12 myotubes increased S1P as consequence of increased expression and activity of SK1 (Hu et al., 2009). Very recently, in the context of free fatty acid oversupply it has been shown that SK1 is regulated at transcriptional level by the transcription factor PPARα in C2C12 cells (Ross et al., 2013). Moreover, specifically in skeletal muscle palmitate treatment induced the expression of IL-6 and its downstream signaling in a SK1- and S1P_3_-dependent manner. IL-6 expression, which is up-regulated in diet-induced obese mice, was attenuated in SK1 null mice. IL-6 produced by skeletal muscle is the prototype of an emerging class of cytokines named myokines which mediate the cross-talk between muscle and other tissues. The role of IL-6 and its signaling in skeletal muscle of obese mice remain unaddressed in this study. Since several studies have reported that muscular IL-6 has a role in metabolism rather than in inflammation, working as an energy sensor and as a lipolytic factor (Pedersen and Febbraio, 2012), the role of SK in the regulation of IL-6 expression and release by skeletal muscle should also be therefore further investigated in other experimental settings.

## Conclusion

Overall, the here reported experimental findings highlight the fundamental role of S1P signaling axis in skeletal muscle biology. Future studies will confidently dissect in detail the molecular mechanisms that regulate S1P metabolism and S1PR expression in the various aspects of skeletal muscle biology and hopefully they will bring to characterize the therapeutic potential of S1P signaling pathway in skeletal muscle diseases.

### Conflict of interest statement

The authors declare that the research was conducted in the absence of any commercial or financial relationships that could be construed as a potential conflict of interest.
